# *Helicobacter pylori* Infection among Patients Undergoing Upper Gastrointestinal Endoscopy in a Tertiary Care Centre

**DOI:** 10.31729/jnma.8393

**Published:** 2024-01-31

**Authors:** Pasanda Sharma, Samyog Adhikari, Shreesuna Katila, Aashra Bajracharya, Nidhi Bohara, Sujan Pathak, Priyanka Poudel, Prakash Sapkota

**Affiliations:** 1 Department of Internal Medicine, Kathmandu University School of Medical Sciences, Dhulikhel, Kavrepalanchok, Nepal; 2 Kathmandu University School of Medical Sciences, Dhulikhel, Kavrepalanchok, Nepal

**Keywords:** *endoscopy*, *Helicobacter pylori*, *upper gastrointestinal tract*

## Abstract

**Introduction:**

*Helicobacter pylori* is a gram-negative gut bacterium associated with dyspepsia, peptic ulcer disease, and gastric cancer, whose prevalence is still common in developing countries. Upper gastrointestinal endoscopy is the gold standard, first-line investigation for evaluating gastrointestinal disorders. The aim of the study was to find out the prevalence of *Helicobacter pylori* infection among patients undergoing upper gastrointestinal endoscopy in a tertiary care centre.

**Methods:**

A descriptive cross-sectional study was conducted in a tertiary health care centre among the patients undergoing upper gastrointestinal endoscopy from 5 January 2020 to 5 January 2023 after obtaining ethical approval from the Institutional Review Committee. Convenience sampling method was used. The point estimate was calculated at a 99% Confidence Interval.

**Results:**

Among 1,975 patients, *Helicobacter pylori* infection was found in 561 (28.41%) (25.79-31.03, 99% Confidence Interval). The indication for upper gastrointestinal endoscopy was mostly dyspepsia 256 (45.68%) followed by abdominal pain 205 (36.54%). The most common endoscopic finding was gastritis 445 (79.32%) followed by hiatal hernia 93 (16.58%). The commonest biopsy finding was chronic active gastritis 478 (85.20%).

**Conclusions:**

The prevalence of *Helicobacter pylori* infection among patients undergoing upper gastrointestinal endoscopy was found to be similar to other studies done in similar settings. The persistence of *H. pylori* emphasizes the need of continuous research to address ever evolving *H. pylori* infections and resistance that are developing against available treatment modalities.

## INTRODUCTION

*Helicobacter pylori (H. pylori)* is a gram-negative gut bacterium associated with dyspepsia, peptic ulcer disease (PUD), and gastric cancer.^1^ *H. pylori* is transmitted by feco-oral route and has such a high prevalence with 80% of the population being H *pylori* positive in developing countries with lower socioeconomic status, overcrowding and poor sanitation.^2,3^ There has been a decrease in *H. pylori* infection with the improvement in living standards in developed countries however it is still highly prevalent in developing countries.^2^ Childhood and early adults are at greatest risk for *H. pylori* infection.^3^ It results in a cascade of diseases including gastritis, atrophy, intestinal metaplasia, dysplasia, and ultimately cancer.^4^

Upper gastrointestinal (GI) endoscopy is the gold standard and first-line investigation for evaluating GI symptoms providing an in-depth microscopic assessment of GI mucosa than any other modality.^5^

The aim of the study was to find out the prevalence of *H. pylori* infection in patients undergoing upper gastrointestinal endoscopy in a tertiary care centre.

## METHODS

This descriptive cross-sectional study was conducted among the patients undergoing upper GI endoscopy in the Gastro-enterology Department of Internal Medicine in Kathmandu University School of Medical Sciences, Dhulikhel hospital, Kavrepalanchok, Nepal after obtaining ethical approval from the Institutional Review Committee of Kathmandu University School of Medical Sciences (Reference number: IRC-KUSMS 38/2021). Data from 5 January 2020 to 5 January 2023 was collected between 12 December 2022 to 5 March 2023 from medical records. Patients above 18 years undergoing upper GI endoscopy for dyspepsia, pain abdomen, dysphagia, anorexia, laryngopharyngeal reflux disorder (LPRD), and for screening purposes were included in the study. Patients with upper GI bleeding due to varices, those with previously diagnosed malignant ulcers, and patients having missing or incomplete data on the medical records of the hospital were excluded. A convenience sampling method was used. The sample size was calculated using the following formula:


$$\begin{array}{ll} \text{n} & ={\text{Z}}^{2}\times \frac{\text{p}\times \text{q}}{{\text{e}}^{\text{2}}}\\ & =2.576^{2}\times \frac{0.50\times 0.50}{0.03^{2}}\\ & =1,843 \end{array}$$

Where,

n = minimum required sample size
Z = 2.576 at 99% Confidence Interval (CI)
p = prevalence taken as 50% for maximum sample size calculation
q = 1-p
e = margin of error, 3%

The calculated sample size was 1,843. However, a total of 1,975 individuals having upper GI endoscopy were included in the study.

The patient underwent an upper gastrointestinal endoscopy using Olympus gastroscopes(GIF-Q160/GIF-Q165/GIF-H185/GIF-1TQ160/GIF-1T140) following an overnight period of fasting during which patients refrained from eating any kind of food and drinking any kind of beverages. Proton pump inhibitor (PPI) was discontinued for 3 days before the procedure. Among the patients meeting the inclusion criteria, biopsy samples for the presence of *H. pylori*were taken from all the patients undergoing the endoscopy. Biopsy samples were taken from the corpus, antrum, and incisura of the stomach. It was preserved in formalin and sent for histopathological examinations. In the pathology laboratory, the sample was stained with Giemsa stain for better yielding of organisms. After examination of the slide under the microscope by a pathologist, the presence and absence of *H. pylori* were determined. If *H. pylori* was present, it was said to be positive and if absent, it was said to be negative.

Data were entered and analyzed using IBM SPSS Statistics version 25.0. The point estimate was calculated at a 99% CI.

## RESULTS

**Figure 1:**
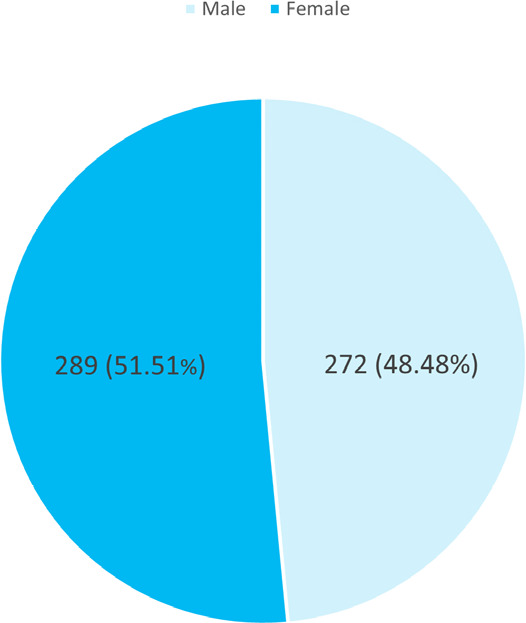
Gender-wise distribution (n= 561)

Among 1975 patients undergoing upper GI endoscopy, the prevalence of *H. pylori* infection was 561 (28.41%) (25.79-31.02, 99% CI) patients. The mean age was 43.1±15.27 years. In addition, 289 (51.51%) were males and 272 (48.48%) were females (Figure 1).

**Figure 2:**
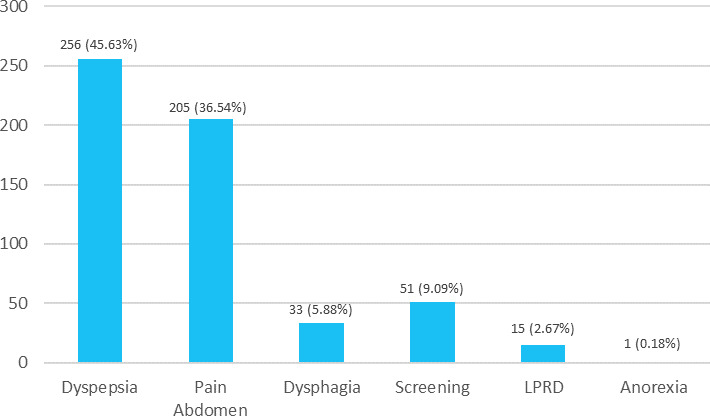
Indications of *H. pylori*-positive patients undergoing UGI endoscopy (n= 561).

A total of 256 (45.63%) patients had dyspepsia, 205 (36.54%) had abdominal pain, 33 (5.88%) had dysphagia, 15 (2.67%) had LPRD and 1 (0.18%) patient had anorexia. Also, 51 (9.09%) patients underwent upper GI Endoscopy for screening purposes (Figure 2).

On upper GI endoscopy of *H. pylori*-positive patients, gastritis was seen in 445 (79.32%), and hiatal hernia in 93 (16.58%) (Table 1).

**Table 1:** Endoscopic findings of *H. pylori*-positive patients (n= 561).

Endoscopic Findings	n (%)
Gastritis	445 (79.32)
Hiatal hernia	93 (16.58)
Duodenal ulcer	47 (8.38)
Oesophagitis	41 (7.31)
Gastric ulcer	31 (5.53)
Gastric polyp	8 (1.43)
Others	36 (6.42)

**Figure 3:**
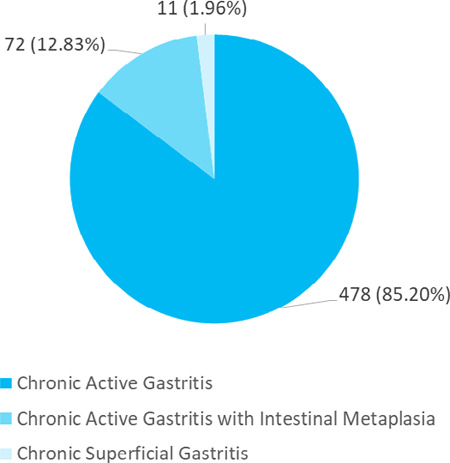
Biopsy findings in *H. pyloi*-positive patients (n= 561).

Majority of the patients had chronic active gastritis 478 (85.20%) on biopsy findings (Figure 3).

## DISCUSSION

Among 1,975 patients undergoing upper GI endoscopy, the prevalence of *H. pylori* infection was 561 (28.41%). The prevalence of a past study conducted in a similar setting showed the prevalence of 23.9%.^6^ Similarly, past studies done in similar settings also showed the prevalence of 38.0%.^7,8^

The adherence and colonization of *H. pylori* are found to be related to genetic alterations, virulence factors expression, and diverse adaptive mechanisms with the release of several effector proteins/toxins.^9^ With a narrow host range, new infections are thought to occur as a consequence of direct human-to- human transmission via genetic predisposition or interpersonal pathways such as the gastro-oral, the oral-oral and the faecal-oral routes.^10^

Accounting for about 80% prevalence in developing countries and 20-50% in industrialised countries, our study focused on the patients undergoing gastroscopy in a tertiary care centre for the indications and findings as well as the *H. pylori* status.^11^ Analyzing the records of 1975 patients meeting inclusion criteria, the prevalence of *H. pylori* in our study was approximately 28.4% with a nearly equal ratio in male and female, unlike our findings, several other studies from Nepal even revealed the ranging prevalence of *H. pylori* to be approximately 16% to 70%, where one study showed equal prevalence in male and female.^12,13^

The diagnostic procedures for *H. pylori* infection have been indicated in the case of dyspepsia as well as in cases of gastric and duodenal ulcers, gastric cancer, regular use of nonsteroidal anti-inflammatory drugs (NSAIDs), mucosa-associated lymphoid tissue (MALT) lymphoma, Barrett's oesophagus, gastroesophageal reflux disease and oesophagal cancer.^4,12^ Similar to this, dyspepsia was the most common indication followed by abdominal pain and a few screening procedures in our study as well. Diagnosis of *H. pylori* infection can be done by noninvasive methods or by endoscopic biopsy of the gastric mucosa according to the clinical setting.^11^ As endoscopic biopsy is also cost-effective with high sensitivity and specificity of approximately 85-100% and is suitable for our hospital setting it was applied in our research.^5^

The total rate of colonization of *H. pylori* in our setting was highest in duodenal ulcers followed by gastric ulcers and gastritis. A 2014 study done in Nepal also showed similar findings whereas another study done showed the complete opposite.^12,14^ In the case of the non-neoplastic lesion found on our biopsy reports, chronic active gastritis was the most commonly found followed by chronic active gastritis with intestinal metaplasia. Another study conducted ten years ago also presented similar findings as ours.^13^ While analyzing the data, we found most of the endoscopic findings revealed positive *H. pylori* cases more in gastritis followed by hiatal hernia, gastroduodenitis, duodenal ulcer, and lax lower esophageal sphincter respectively. In one study, the most common endoscopic findings were erythematous antral gastritis followed by erosive gastritis, duodenal ulcer, and gastric ulcer.^6^ H pylori treatment may not resolve dyspeptic symptoms in a broader aspect but with effective eradication, the risk of peptic ulcer or gastric cancer in the long run will lower.^15^ Thus, although sequential therapy can be recommended for the treatment to eradicate *H. pylori*, in our settings standard triple-drug regimen is still primarily prescribed but second-line therapies should also be considered with the understanding of variable resistance and treatment efficacy.

There are a few limitations in our study. As the study was carried out at a single tertiary care facility, this might not accurately reflect the large geographical area. Multicenter studies with larger sample sizes are needed to provide a more comprehensive picture of the *H. pylori* status.

## CONCLUSIONS

The prevalence of *H. pylori* infection among patients undergoing upper GI endoscopy is similar to other studies done in similar settings.
